# Prognostic value of peripheral blood natural killer cells in colorectal cancer

**DOI:** 10.1186/s12876-020-1177-8

**Published:** 2020-02-07

**Authors:** Yan-ping Tang, Ming-zhi Xie, Ke-zhi Li, Ji-lin Li, Zheng-min Cai, Bang-li Hu

**Affiliations:** 1 Department of Research, Guangxi Medical University Cancer Hospital, 71 Hedi Road, Nanning 530021, China, Nanning, 530021 Guangxi People’s Republic of China; 2 Department of Chemotherapy, Guangxi Medical University Cancer Hospital, Nanning, 530021 Guangxi People’s Republic of China

**Keywords:** Colorectal cancer, NK cells, Peripheral blood, Prognosis

## Abstract

**Background:**

The association between natural killer (NK) cells and survival in colorectal cancer (CRC) patients remains controversial. This study aimed to clarify the prognostic value of peripheral blood NK cells in CRC patients.

**Methods:**

A total of 447 CRC patients who underwent radical surgery and chemotherapy were retrospectively analyzed. Cox regression analyses were used to identify independent prognostic indicators. Correlation between NK cell percentage and other clinicopathological features (gender, age, histological grade, tumor stage, immune cells, and inflammatory indicators) was analyzed. The prognostic values of the combinations of NK cell percentage and other clinicopathological features were also determined.

**Results:**

Multivariate Cox regression analysis revealed that NK cell percentage in the peripheral blood was an independent prognostic indicator in CRC patients. A higher percentage of NK cells indicated a longer survival time than a lower percentage. NK cell percentage was positively correlated to the T and B lymphocyte counts and negatively correlated to the patients’ age and albumin levels. With an area of 0.741 under a receiver operating characteristic curve, NK cells have a moderate predictive value for 3rd-year survival in CRC. This area increased to 0.851 by combining NK cell percentage with the B lymphocyte count. Elderly patients and those at an advanced clinical stage presented a lower percentage of NK cells than younger patients and those at an early clinical stage.

**Conclusions:**

This study demonstrated that NK cells in the blood were an independent predictor of survival in CRC patients, and the combined count of NK cells and B lymphocytes could increase the prognostic value.

## Background

Colorectal cancer (CRC) is a common cause of cancer-related mortality [1]. Unfortunately, at the initial diagnosis, about one-fourth of the CRC patients are at an advanced stage [2]. Surgery and chemotherapy are the major treatment approaches for CRC; however, their effect is unsatisfactory in patients with distant metastases. This poor effect can be attributed to a suppressed immune system that lacks an effective anti-tumor activity [3, 4]. The human immune system is composed of many immune cells. Among these, the T lymphocytes, B lymphocytes, and natural killer (NK) cells form a vital component of the host antitumor immune response. These cells play crucial roles in the initiation, development, and progression of cancers [5–7]. They are also considered as potential targets in immunotherapy and clinical biomarker research [8].

The NK cells are the cytotoxic lymphocytes of the innate immune system. They kill target cells without prior antigen presentation and are involved in coordinating the adaptive immune response [9–11]. This suggests that these cells could serve as biomarkers for monitoring the immune system. The prognostic value of NK cells has been described in some cancers. For example, a low count of NK cells in the peripheral blood was found to be associated with worse outcomes in follicular lymphoma patients who were receiving an antibody-based therapy [12]. While the prognostic value of NK cells in CRC patients has also been reported, these results are inconsistent [13–15], probably due to a small sample size or some other factors. Therefore, this study aimed to examine whether NK cells could serve as an independent prognostic indicator of survival in CRC patients who underwent radical surgery and adjuvant chemotherapy.

## Methods

### Selection of CRC patients

The data of CRC patients who underwent surgery and chemotherapy at the Guangxi Medical University Cancer Hospital between January 2015 and October 2018 was retrospectively analyzed. The inclusion criteria consisted of the following: (1) CRC was confirmed histologically; (2) the patients underwent surgical R0 or R1 resection; and (3) the patients underwent chemotherapy using FOLFOX- or Capox-based regimens. Patients with autoimmune, inflammatory, and severe hematological diseases and major organ failure were excluded. This study was conducted in accordance with the ethical guidelines of the 2008 Declaration of Helsinki and was approved by the ethics committee of the Guangxi Medical University Cancer Hospital.

### Data collection

Data concerning the clinicopathological features of the selected CRC patients were extracted. This included their age, gender, tumor location, differentiation degree, and tumor node metastasis (TNM) stage [American Joint Committee on Cancer (AJCC criteria) 7th edition] [16]. Blood parameter data, including the levels of tumor biomarkers (such as CEA, CA125, CA153, and CA199); percentages of T lymphocytes, B lymphocytes, and NK cells; and levels of C-reactive protein (CRP) and high-sensitive CRP (hsCRP) were extracted. All blood samples were collected before the treatment. The platelet-lymphocyte ratio, neutrophil-lymphocyte ratio, and C-reactive protein/albumin ratio (CAR), which are reported to be associated with CRC prognosis [17, 18], were calculated. The CRC patients were followed up regularly until October 2018 or until death. The overall survival (OS) was calculated from the date of the surgery to the date of death or the last follow-up.

### Flow cytometry assay for T and B lymphocytes and NK cells

Before the surgery, fresh blood samples were collected into heparinized tubes and analyzed immediately after staining with monoclonal antibodies. Lymphocytes were gated according to the CD45 or side scatter dot plots. In the present study, T cell subsets were defined as CD3+/CD4+ lymphocytes, B cells were defined as CD19+ lymphocytes, and NK cells were defined as CD3−/CD16+/CD56+ lymphocytes (BD Biosciences, Franklin Lakes, NJ, USA). The flow cytometry assay for lymphocytes and NK cells was performed according to a previously described protocol [19]. Flow cytometry (BD Biosciences) was used to detect the labeled cells and analyze the results.

### Statistical analysis

Statistical analyses were performed using SPSS (version 21.0) and R (version 3.5.1). All statistical tests were two-sided and *p*-values <0.05 were considered as statistically significant. The Kruskal—Wallis test was used to examine the statistical differences among three groups or more. The Mann—Whitney *U*-test and the Student’s *t*-test were used to compare continuous variables between two groups when appropriate. The Kaplan—Meier curve and the log-rank test were used to determine the differences in the survival rates between two groups. We used continuous data for NK cells, other immune cells, and inflammatory indicators in the Cox regression analyses. A univariate Cox regression analysis was performed using the patients’ clinicopathological features such as gender, age, histological grade, tumor stage, immune cells, and inflammatory indicators. A multivariate Cox regression analysis was performed to identify the independent predictors of survival in CRC patients. The prognostic value of NK cells was evaluated from a receiver operating characteristic (ROC) curve and the area under the ROC curve (AUC). Pearson correlation analysis was used to determine the association between the NK cell percentage and other clinicopathological features. The *p*-value was adjusted for multiple comparisons using the Benjamini-Hochberg correction.

## Results

### Clinical characteristics of the study objects

A total of 447 CRC patients who underwent surgery and chemotherapy were selected after applying the inclusion and exclusion criteria. Among these, 224 suffered from colon cancer and 223 from rectal cancer. Around 12, 124, 278, and 33 patients were diagnosed in 2015, 2016, 2017, and 2018, respectively. While most patients underwent an R0 resection, 48 stage IV patients (with tumor distant metastasis) underwent an R1 resection. The median age of the patients was 59.5 years. The median follow-up was 24 months (range: 16–28 months). Around 387 patients were alive, while 60 patients died during the follow-up period. The patients’ details are given in Table 1.

**Table 1:** Clinical characteristics of the study populations

Variables	Value
Age	59.5 ± 13.08
Gender	
Male/Female	258/189
Location	
Colon/Rectal	224/223
Histological grade	
High/Middle/Low	53/86/308
T stage	
T1/T2/ T3/ T4/ TX	7/52/102/251/35
N stage	
N0/N1/N2/N3	152/160/89/46
M stage	
M0/M1/MX	279/157/11
White blood cells	6.36 (5.14–8.10)
Hemoglobin	118.5 (102.2–130.1)
Platelet	271.3 (215.5–339.5)
Neutrophil	3.85 (2.93–5.23)
Lymphocyte	1.62 (1.27–2.03)
ALB	36.60 (33.90–39.30)
CEA	3.11 (1.21–17.71)
CA125	10.80 (7.10–25.15)
CA153	8.50 (4.95–12.95)
CA199	16.60 (8.15–39.55)
T lymphocyte	65.10 (59.70–71.25)
B lymphocyte	12.70 (9.40–16.20)
NK	15.60 (11.45–20.20)
CRP	3.31 (1.34–11.29)
hsCRP	0.83 (0.42–3.85)
NLR	2.29 (1.65–3.43)
PLR	165.42 (119.81–242.77)
CAR	0.09 (0.03–0.32)

*NK* Natural killer, *CEA* Carcinoembryonic antigen, *ALB* Albumin, *CRP* C-reactive protein, *hsCRP* High sensitivity C-reactive protein, *NLR* Neutrophil-lymphocyte ratio, *PLR* Platelet lymphocytes ratio, *CAR* CRP/ALB ratio

### Univariate and multivariate cox regression analyses using clinicopathological features

Univariate analysis revealed that CA125, percentages of T and B lymphocytes and NK cells, CRP levels, clinical stage, and CAR were the independent prognostic factors for of survival in the CRC patients. Multivariate analysis revealed that CA125, T lymphocytes, B lymphocytes, NK cells, and CRP levels were the independent prognostic factors for survival in the CRC patients (Table 2).

**Table 2:** Univariate and multivariate Cox regression analysis for the baseline characteristics

	Univariate Cox regression analysis			Multivariate Cox regression analysis		
	*P*-value	HR	95%CI	*P*-value	HR	95%CI
Age	0.877	1.002	0.982–1.021			
Gender	0.836	0.947	0.569–1.579			
Neutrophil	0.594	0.968	0.858–1.092			
Lymphocyte	0.544	0.925	0.639–1.266			
Platelet	0.533	1.001	0.999–1.003			
ALB	0.333	1.026	0.974–1.081			
CEA	0.108	0.996	0.992–1.001			
CA125	0.032	0.986	0.972–0.999	0.003	0.976	0.961–0.992
CA153	0.538	0.993	0.96–1.022			
CA199	0.375	0.982	0.998–1.001			
T lymphocyte	0.039	0.979	0.959–0.999	<0.001	0.951	0.928–0.974
B lymphocyte	<0.001	0.755	0.697–0.816	<0.001	0.793	0.736–0.854
NK	0.004	0.938	0.898–0.979	0.003	0.932	0.889–0.976
CRP	<0.001	1.019	1.014–1.025	0.038	1.085	1.005–1.172
hsCRP	0.349	1.026	0.972–1.083			
Histological grade						
High	–	–	–			
Middle	0.639	0.789	0.294–2.119			
Low	0.768	0.669	0.384–2.579			
T stage						
T1 + T2	–	–	–			
T3 + T4	0.165	1.200	0.515–2.795			
N stage	0.758	1.099	0.603–2.001			
N0	–	–	–			
N1 + N2 + N3	0.449	1.192	0.757–1.182			
M stage						
M0	–	–	–			
M1	0.383	1.090	0.711–1.672			
MX	0.692	1.074	0641–1.799			
Clinical stage						
I + II	–	–	–			
III + IV	0.033	1.992	1.058–3.749	0.139	2.651	0.338–12.178
NLR	0.849	0.995	0.947–1.046			
PLR	0.261	1.001	0.999–1.002			
CAR	< 0.001	1.624	1.397–1.889	0.586	1.390	0.425–4.544

*NK* Natural killer, *CEA* Carcinoembryonic antigen, *ALB* Albumin, *CRP* C-reactive protein, *hsCRP* High sensitivity C-reactive protein, *NLR* Neutrophil-lymphocyte ratio, *PLR* Platelet lymphocytes ratio, *CAR* CRP/ALB ratio

### Survival analysis and prognostic value of NK cells in CRC patients

**Fig. 1:**
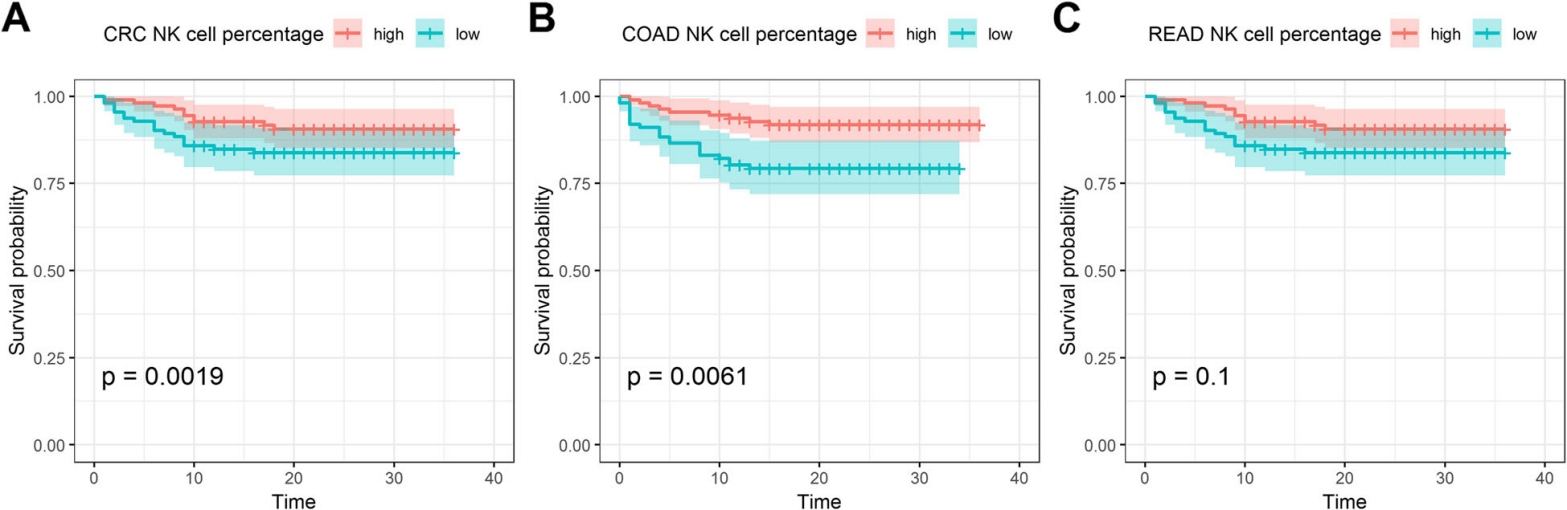
**a** Kaplan-Meier curve for the percentage of NK cells in CRC patients; **b**: Kaplan-Meier curve for the percentage of NK cells in colon cancer patients; **c**: Kaplan-Meier curve for the percentage of NK cells in rectal cancer patients

Using the median percentage value of NK cells, we found that patients with a lower percentage of NK cells had shorter survival times than those with a higher percentage. We also compared the survival times of NK cells in colon and rectal cancer patients. Colon cancer patients with a lower percentage of NK cells had shorter survival times than those with a higher percentage; however, the same was not observed for rectal cancer (Fig. 1). Furthermore, in the 60 dead and 387 living patients, we analyzed the association between NK cell percentage and the year of diagnosis; the NK cell percentage was only found to be associated with the survival of colon cancer patients in 2016 when 124 patients were diagnosed (*P* = 0.009, Additional file 1: Figure S1). This suggests that the sample size affects the prognostic value of NK cells.

**Fig. 2:**
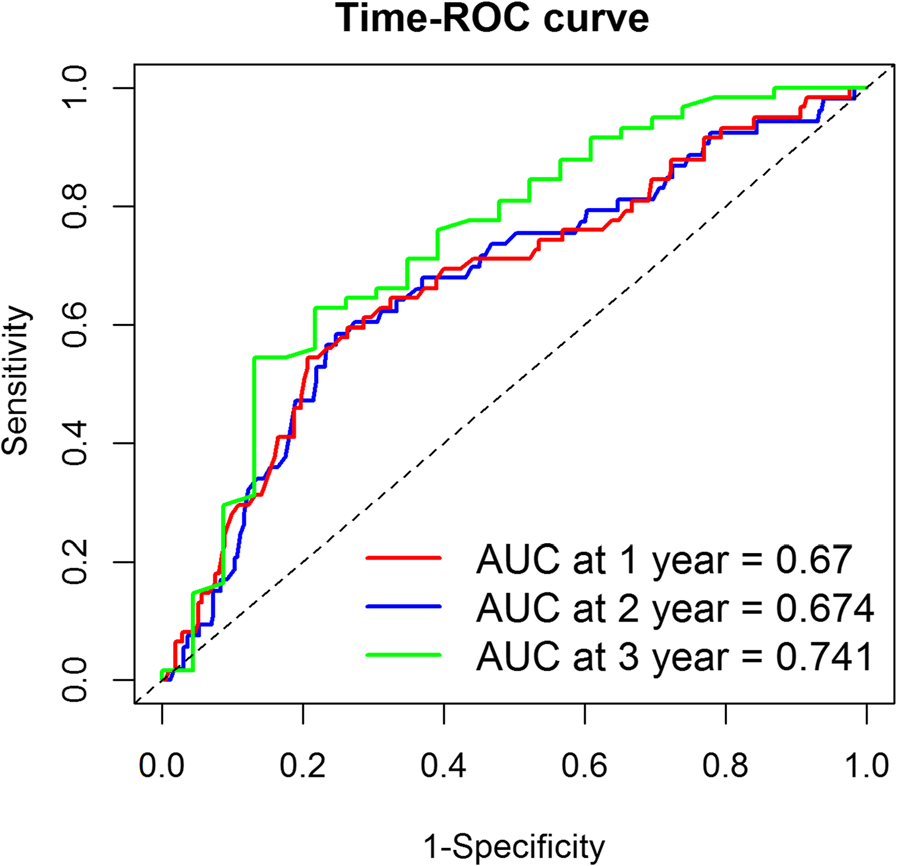
Time ROC curve for the prognostic value of the percentage of NK cells on the 1-, 2-, 3- years survival in CRC patients

We then determined the prognostic value of NK cells in CRC patients using the results from the multivariate Cox regression analysis and found the percentage of NK cells to be a satisfactory predictor for the 1st, 2nd, and 3rd year of survival, with an AUC of 0.670, 0.674, and 0.741, respectively (Fig. 2).

### Correlation between NK cell percentage and clinicopathological features

Pearson correlation analysis revealed that NK cell percentage was positively correlated to T and B lymphocyte counts and negatively correlated to the patients’ age and albumin (ALB) levels (Table 3).

**Table 3:** Significant correlation of NK cell and the clinicopathological features

NK cells	r	*P*-value
Age	−0.110	0.019
ALB	−0.137	0.003
T lymphocyte	0.221	0.001
B lymphocyte	0.115	0.015

### Association of NK cells with the clinicopathological features

The association of NK cells with the patient’s age, gender, histological grade, TNM stage, and clinical stage was analyzed. As shown by Table 4, older patients and those at an advanced clinical stage presented a lower percentage of NK cells than younger patients and those at an early clinical stage (*P* < 0.05). However, no significant difference was found between the genders, cancer differentiation, and TNM stage (*P* > 0.05).

**Table 4:** Association of NK cell with the clinicopathological features

Variable	Percentage of NK cell	*P*-value
Age		
> 60	18.24 ± 7.49	0.013
≤ 60	15.13 ± 7.76	
Gender		
Male	17.27 ± 7.98	0.068
Female	15.96 ± 7.08	
Location		
Colon	16.84 ± 8.04	0.742
Rectal	16.60 ± 7.21	
Histological grade		
High	16.64 ± 7.56	0.784
Middle	17.90 ± 7.62	
Low	16.40 ± 7.63	
T stage		
T1 + T2	16.73 ± 7.82	0.920
T3 + T4	16.64 ± 6.29	
N stage		
N0	16.97 ± 6.90	0.593
N1 + N2 + N3	16.59 ± 7.99	
M stage		
M0	17.05 ± 7.45	0.473
M1	16.52 ± 7.74	
Clinical stage		
I + II	19.25 ± 7.06	0.029
III + IV	16.47 ± 7.88	

### Joined effect of NK cells and clinicopathological features in predicting patient prognosis

**Fig. 3:**
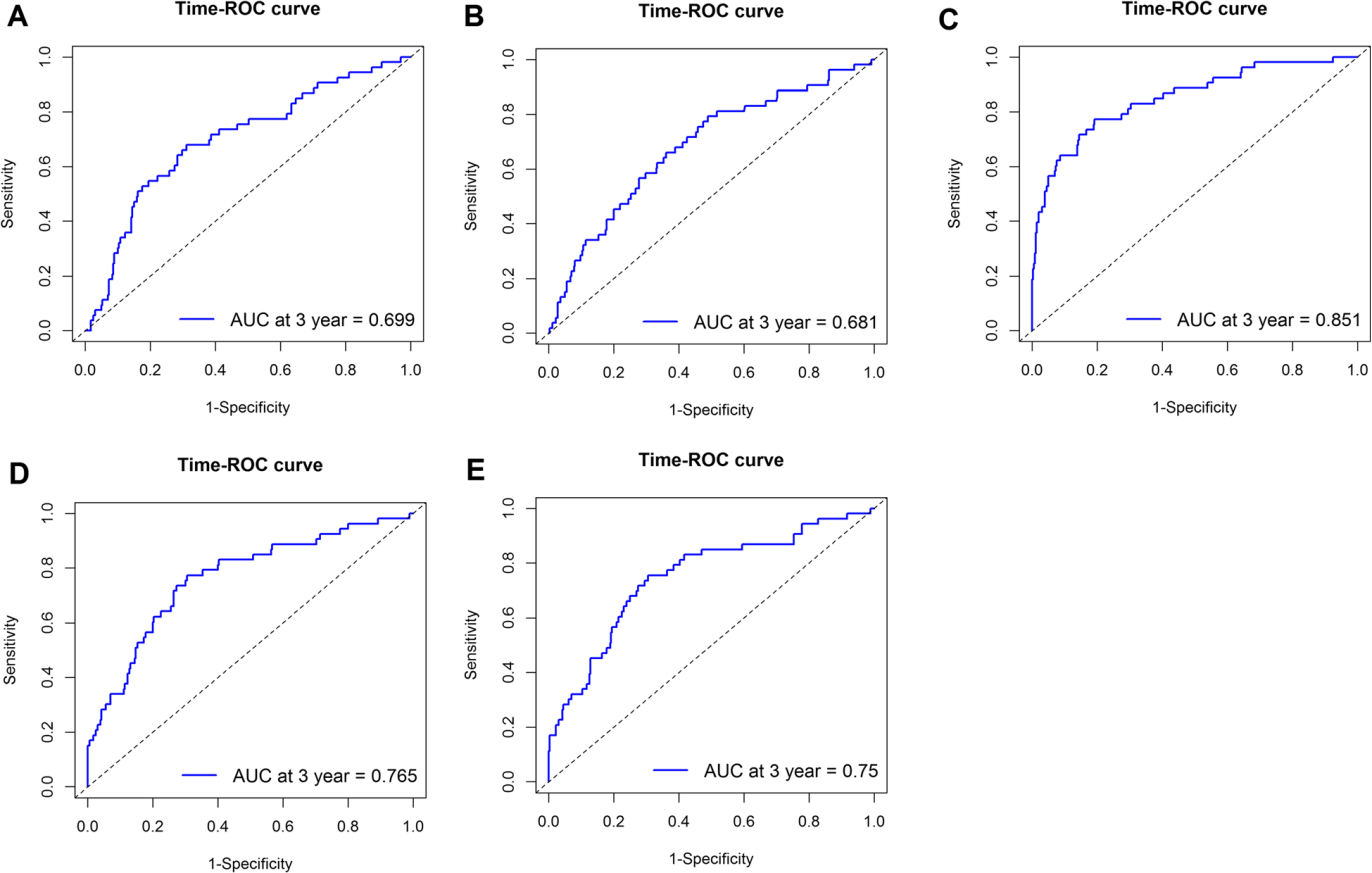
Prognostic value of the percentage of NK cells combining clinicopathological features. **a**: NK cells combining CA125; **b**: NK cells combining T lymphocyte; **c**: NK cells combining B lymphocyte; **d**: NK cells combining CRP; **e**: NK cells combining CAR

In order to improve the prognostic value of NK cells, we joined NK cell percentage with the clinicopathological features that were significantly associated with CRC prognosis i.e., CA125, T and B lymphocyte counts, CRP levels, and CAR. A combination of NK cell percentage and B lymphocyte count was found to achieve the highest prognostic value for predicting the 3rd year survival, with an AUC of 0.851. The next highest prognostic value was offered by a combination of NK cell percentage and CAR, followed by combinations with CRP, T lymphocyte count, and CA125 (Fig. 3).

## Discussion

In this study, we included 447 CRC patients who underwent surgery and chemotherapy. NK cells originate in the bone marrow from the CD34 + Lin-CD45RA + CD10+ common lymphoid progenitor cells [20]. They represent a crucial component of the antitumor innate immune response and perform cytotoxic functions through secreting several cytokines and chemokines [21]. In case of cancer, a decrease in the NK cell count is often observed [22]. NK cell count is reported to be associated with the survival of CRC patients. Coca et al. analyzed the data of 157 CRC patients and found that patients with an extensive CD57+ NK cells intratumoral infiltration have a longer survival than those with a low infiltration [23]. Conversely, Sandel et al. analyzed the data of 88 CRC patients and showed that NK cell infiltration had no effect on the survival of CRC patients [24].

In our study, the percentage of NK cells in the peripheral blood was an independent prognostic indicator in CRC patients. This was in agreement with the report by Coca et al. [23]. However, when the data was divided according to the colon and rectal cancer patients, a significant difference in the survival time was only observed for colon cancer and not rectal cancer. This suggests that NK cells may have a different effect on survival in these two cancers.

It is well known that immunity decreases with aging and the number of immune cells in cancer patients changes with the development of the disease [25, 26]. In the present study, we found that the circulating NK cells were positively correlated to the T and B lymphocytes, which are important immune cells of the human immune system [5–7]. Furthermore, we observed that the NK cell percentage was negatively correlated to the patient’ age and ALB levels, suggesting that this prognostic indicator may collaborate with other immune indicators in CRC and decrease with aging. Similarly, the association between NK cells and other clinicopathological features further confirmed this relationship between NK cell percentage and the patients’ age and clinical stage.

The present study determined the prognostic value of NK cells in CRC patients; the value was moderate for the prediction of the 1st, 2nd, and 3rd year of survival. Previous studies have reported that blood CEA, CA199, and B lymphocyte counts are associated with survival in CRC patients [27–29]. We confirmed these associations in the present study. We observed that a combination of NK cell percentage with these variables can increase the prognostic value; its combination with B lymphocyte count achieves the highest prognostic value compared to other combinations. Our findings suggested that in CRC patients, a combination of NK cell percentage and B lymphocyte count may serve as a good predictor of survival after surgery and chemotherapy.

Although our study failed to show an association between NK cells and survival in rectal cancer patients, Coyle et al. found that a high percentage of NK cells in cancerous rectal tissues improved survival in these patients. We speculated that our test sample and method may have led to different results. Studies have reported that the percentage of NK cells in the peripheral blood was not inconsistent between the core of the lung cancer [30] and cervical cancer [31]. We also observed that the TNM stage had no significant association with the survival of CRC patients. We believe that the length of follow-up may be one of the factors. Because most of the patients underwent an R0 resection, the median follow-up was 24 months, which was shorter than the follow-up of these patients.

Compared with previous studies, the present study included a larger CRC patient cohort; therefore, more reliable results could be achieved. However, this study had some limitations. First, the study’s retrospective design may have led to selection bias, thereby reducing the robustness of the results. Second, the follow-up period (median follow-up: 24 months) in this study was relatively shorter. The prognostic value of NK cells for a longer survival requires further investigation. Third, NK cells can be divided into CD56^dim^ and CD56^bright^ cells [32], which have different antitumor activities; however, this study did not analyze these two subsets, because our laboratory did not separate them during the test. Fourth, the length of follow-up for R0 resection patients was relatively shorter; we did not collect data on disease-free survival or recurrence-free survival, which are more appropriate for describing the association in patients who underwent an R0 resection. Therefore, future studies should address these issues in order to confirm the role of NK cells in CRC.

## Conclusions

The present study demonstrated that the percentage of NK cells in the blood was an independent predictor of survival in CRC patients, and a combination of NK cell percentage and B lymphocyte count had a high prognostic value. However, future studies are needed to verify our results due to the abovementioned limitations.

## Supplementary information

**Additional file 1: Figure S1.** Association beween NK cell percentage and the year of diagnosis. A. Kaplan-Meire curve for the percentage of NK cells in CRC patients in 2015; B: Kaplan-Meire curve for the percentage of NK cells in CRC patients in 2016; C: Kaplan-Meire curve for the percentage of NK cells in CRC patients in 2017; D: Kaplan-Meire curve for the percentage of NK cells in CRC patients in 2018.

## Data Availability

The data used to support the findings of this study are included within the article. (Table 1).
