# Supplementary figures and images for: Prognostic value of peripheral blood natural killer cells in colorectal cancer
Source: BMC Gastroenterol. 2020 Feb 7;20:31. doi: 10.1186/s12876-020-1177-8 (PMC7006176; doi:10.1186/s12876-020-1177-8)

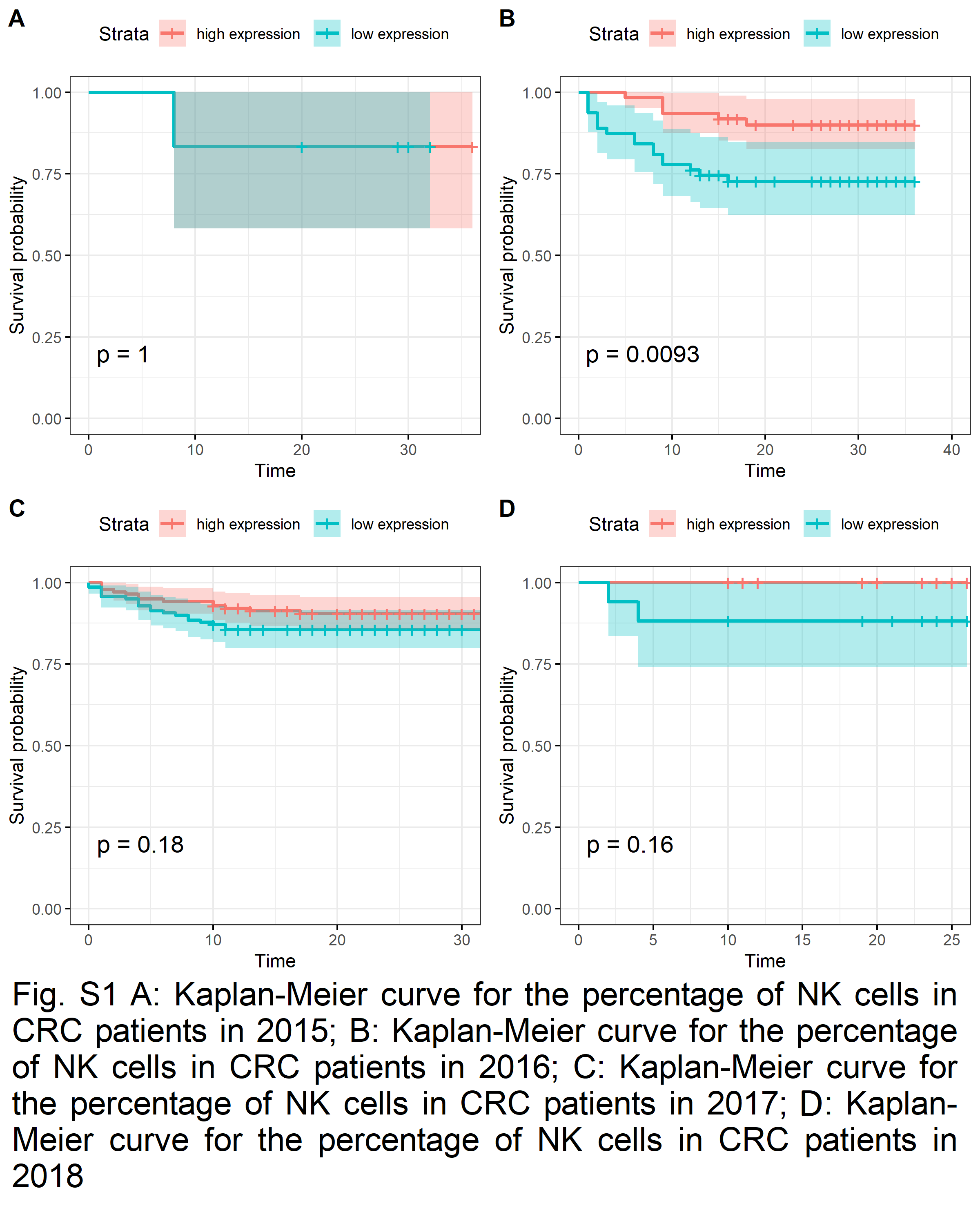

Supplement: Supplementary file 1 — Additional file 1: Figure S1. Association beween NK cell percentage and the year of diagnosis. A. Kaplan-Meire curve for the percentage of NK cells in CRC patients in 2015; B: Kaplan-Meire curve for the percentage of NK cells in CRC patients in 2016; C: Kaplan-Meire curve for the percentage of NK cells in CRC patients in 2017; D: Kaplan-Meire curve for the percentage of NK cells in CRC patients in 2018. [file 12876_2020_1177_MOESM1_ESM.tif]
